# The normalizing properties of intracranial volume across race and sex

**DOI:** 10.1093/braincomms/fcaf271

**Published:** 2025-07-16

**Authors:** Peirong Liu, Dina Zemlyanker, Karthik Gopinath, You Cheng, Yingnan He, David Izquierdo-Garcia, Teresa Gomez-Isla, Sudeshna Das, Shahin Nasr, Kevin N Sheth, Matthew S Rosen, W Taylor Kimberly, Adam de Havenon, Francis X Shen, Juan Eugenio Iglesias

**Affiliations:** Martinos Center for Biomedical Imaging, Massachusetts General Hospital and Harvard Medical School, Charlestown, MA 02129, USA; Department of Electrical and Computer Engineering, Johns Hopkins University, Baltimore, MD 21218, USA; Martinos Center for Biomedical Imaging, Massachusetts General Hospital and Harvard Medical School, Charlestown, MA 02129, USA; Martinos Center for Biomedical Imaging, Massachusetts General Hospital and Harvard Medical School, Charlestown, MA 02129, USA; Department of Neurology, Massachusetts General Hospital and Harvard Medical School, Boston, MA 02114, USA; Department of Neurology, Massachusetts General Hospital and Harvard Medical School, Boston, MA 02114, USA; Martinos Center for Biomedical Imaging, Massachusetts General Hospital and Harvard Medical School, Charlestown, MA 02129, USA; Harvard-MIT Health Sciences and Technology, Massachusetts Institute of Technology, Cambridge, MA 02139, USA; Department of Bioengineering, Universidad Carlos III de Madrid, Madrid, 28903, Spain; Department of Neurology, Massachusetts General Hospital and Harvard Medical School, Boston, MA 02114, USA; Department of Neurology, Massachusetts General Hospital and Harvard Medical School, Boston, MA 02114, USA; Martinos Center for Biomedical Imaging, Massachusetts General Hospital and Harvard Medical School, Charlestown, MA 02129, USA; Center for Brain and Mind Health, Yale School of Medicine, New Haven, CT 06519, USA; Martinos Center for Biomedical Imaging, Massachusetts General Hospital and Harvard Medical School, Charlestown, MA 02129, USA; Department of Neurology, Massachusetts General Hospital and Harvard Medical School, Boston, MA 02114, USA; Center for Brain and Mind Health, Yale School of Medicine, New Haven, CT 06519, USA; Harvard Medical School Center for Bioethics, Harvard Medical School, Boston, MA 02115, USA; Department of Psychiatry, Massachusetts General Hospital, Boston, MA 02114, USA; University of Minnesota, Minneapolis, MN 55455, USA; Martinos Center for Biomedical Imaging, Massachusetts General Hospital and Harvard Medical School, Charlestown, MA 02129, USA; Hawkes Institute, University College London, London WC1V 6LJ, UK; Computer Science and Artificial Intelligence Laboratory, Massachusetts Institute of Technology, Cambridge, MA 02139, USA

**Keywords:** intracranial volume normalization, human brain MRI, volumetry

## Abstract

In volumetric analysis of the human brain with MRI, intracranial volume is an important covariate as it has a strong correlation with the volume of regions of interest in the brain. Therefore, accurate adjustment for intracranial volume (e.g. by division) is essential to mitigate the impact of head size on brain measurements. In this study, we assess the effects of intracranial volume normalization on differences across sex and racial groups, using a diverse cohort with 5977 subjects from three different (self-reported) races. We show that (i) differences in intracranial volume across sex are consistent across race and vice versa; and (ii) intracranial volume normalization almost completely accounts for differences by race and sex. These results suggest that subjects of different sexes and races can be safely aggregated in volumetric studies by normalizing the volume of regions of interest to intracranial volume.

## Teaser

Liu *et al*. report that intracranial volume strongly correlates with brain region volumes in MRI studies. Using a diverse cohort, they show that intracranial volume normalization effectively removes sex and race differences, enabling safe aggregation of subjects across groups in volumetric analyses.

## Graphical Abstract

**Graphical Abstract:**
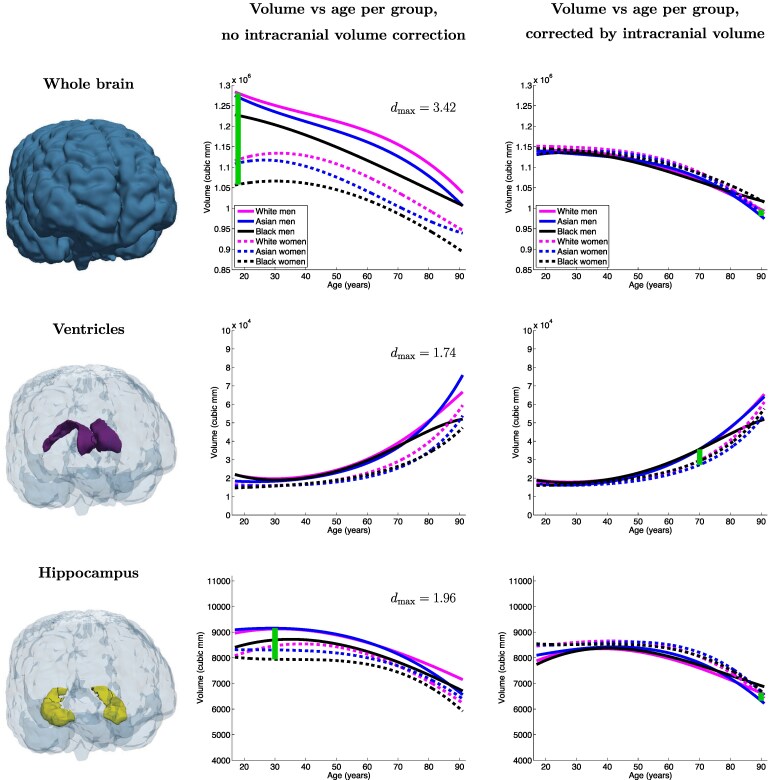

## Introduction

MRI is pivotal in imaging of the human brain *in vivo*, as it provides a non-invasive view into its complex anatomical structure, neural pathways and functional dynamics.^1^ Specifically, morphometric analysis of brain MRI scans enables volumetry of regions of interest (ROIs), which in turn enables the study of structural changes associated with normal aging and various diseases, such as Alzheimer’s disease,^2^ schizophrenia^3^ and traumatic brain injury (TBI).^4^ By measuring alterations in brain volume, researchers can gain insight into disease progression^5^, evaluate treatment efficacy^6^ and identify potential biomarkers,^7^ thus advancing our understanding and management of these disorders.

In conducting volumetric analyses, researchers consider various covariates such as age, sex and other demographic variables.^8^ Among these, intracranial volume (ICV) is particularly important due to its strong correlation with ROI volumes.^9^ Accurate adjustment for ICV is crucial, whether through direct division or as a nuisance variable in general linear models, to account for the influence of head size on brain measurements and ensure precise analysis and interpretation. Even though sex is also commonly included as a covariate in volumetry, the incorporation of ethnicity and race as explanatory variables remains limited.^10^ Reasons include: subjective and potentially inconsistent labelling and measurement of ethnoracial categories; historical misuse of race-based analyses to draw inferences about racial hierarchies; measuring racism and other social determinants of health instead; or interaction with other variables, among others.^11-13^

In this study, we highlight that normalizing for ICV by simple division effectively eliminates most volumetric variations observed across sex and race. We analysed a diverse retrospective cohort with 5977 subjects scanned at Massachusetts General Hospital (MGH) whose scans did not reveal any structural brain abnormalities. Our results show that ICV normalization corrects most of the systematic differences in brain volumes attributed to race and sex.

## Materials and methods

### Data acquisition

In this study, we used MRI scans from 5977 MGH subjects who were scanned at the hospital between 2014 and 2024. These scans were downloaded from the MGH picture archiving and communication system (PACS) under IRB protocol 2015P001915. The selection criteria were: age between 18 and 90 years; presence of non-surgical brain MRI scans; and absence of structural abnormalities, such as tumours, strokes, or traumatic brain injury—which is automatically determined from their clinical data repository by the MGH Research Patient Data Registry system (RPDR). These criteria yielded a cohort that was as close as possible to a control group. Race is self-reported, and is captured in the electronic health record, and automatically extracted into RPDR. We utilized data from all available Asian and Black subjects satisfying the criteria (1799 and 2051, respectively), as well as 2150 White subjects to complete a sample size of *N* = 5977.

We note that data was acquired from various scanners at hospitals affiliated with MGB over the past 10 years, including periods of scanner upgrades. Unfortunately, we do not have detailed or reliable information on the specific scanning platforms. Nevertheless, the SynthSeg+ method has been thoroughly validated in this scenario.^14^

### Statistical analysis

Automated segmentation is performed using SynthSeg+,^14^ which provides segmentations and volumes for 31 ROIs, including the ICV. Each MRI session may consist of a varying number of 3D scans. We utilize SynthSeg+ as described in its original publication and include only those scans that achieve automatic quality control (QC) scores > 0.75. If more than three scans meet this criterion, we select the top three scans with the highest QC scores. The ROI volumes are then averaged from the scans that pass the QC check, or the top three scans when applicable. For subjects with multiple visits, we average the ROI volumes and ages from these different visits to obtain a single estimate and make the analysis independent of the number of visits. This ensures that the analysis accounts for variability in age and scanning quality across multiple sessions. We note that 23 subjects did not have any scans passing the QC criteria, which led to the final sample size of *N* = 5977 (Table 1). ICV correction was achieved by direct division—followed by direct multiplication of the average ICV, which preserves the mean volume of the ROIs after normalization. We note that correction by other widespread method (residual, covariate) did not affect the results (please see further details in the ‘Results’ section and the supplementary materials).

**Table 1:** Number of subjects in our study, categorized by (self-reported) race and sex

Race/sex	Female	Male
Asian	1058 (age: 48.9 ± 18.6)	733 (age: 52.3 ± 18.6)
Black	1213 (age: 52.0 ± 18.5)	833 (age: 53.1 ± 18.1)
White	1179 (age: 55.1 ± 19.0)	961 (age: 55.4 ± 17.7)

When fitting curves, we acknowledge that outliers may arise, either from missegmentations (as SynthSeg+ might occasionally fail), or from the abnormalities that may be missed by RPDR. For this reason, we employ robust statistical analysis. Specifically, we use a Laplacian distribution to model the data, where the location and scale parameters are smooth functions of age. To achieve this, we model the location and scale using B-splines with four control points equally spaced at ages 27, 45, 63 and 81. The fit is optimized with gradient ascent over the log-likelihood function:


(1) $$L(\theta_{\mu },\theta_{b})=\;\overset{N}{\underset{n=1}{\sum }}\;\log\;p[v_{n};\mu (a_{n},\theta_{\mu }),\;b(a_{n},\theta_{b})]$$

where p(x;μ,b) is the probability density function of the Laplace distribution with location *μ* and scale *b*; $v_{n}$ is the volume of the ROI for subject *n*; $a_{n}$ is the age of subject *n*; $\mu (a_{n},\theta_{\mu })$ is a B-spline describing the location, parameterized by $\theta_{\mu }$; and $b(a_{n},\theta_{b})$ is a B-spline describing the scale, parameterized by $\theta_{b}$. Note that the location parameter *μ* represents the median that minimizes the sum of L1 errors, and the 95% confidence interval is (almost exactly) given by three times the scale, i.e. μ±3b.

We compute effect sizes at any given age using the pooled (average) scales:


(2) $$d=\frac{2|\mu_{1}(a;\theta_{\mu_{1}})-\mu_{2}(a;\theta_{\mu_{2}})|}{b_{1}(a;\theta_{b_{1}})+b_{2}(a;\theta_{b_{2}})}$$

where *a* is the age, and the subindices 1 and 2 refer to the two compared groups.

Finally, we note that, for the ventricles, we modelled the logarithm of their volume rather than the volume directly, since the distribution of ventricular volumes at any given age is considerably skewed towards higher values.

## Results

Table 1 summarizes the sample size and demographics of the cohort used in this study, which includes 5977 subjects from three different racial categories: Asian, Black and White. Table 2 displays ICV values categorized by self-reported race and sex. Crucially, this table shows that the differences between sexes are very consistent across races and vice versa: the male/female ICV ratio is 1.119 for Asian subjects, 1.121 for Black subjects and 1.127 for White subjects; whereas the racial ICV ratios are: 1.032, 0.953 and 0.983 for females; versus 1.029, 0.947 and 0.976 for males. We note that the global male/female ratio (1.12) is highly consistent with previous studies.^15^

**Table 2:** ICV values categorized by race and sex (in litres)

Race/sex	Female	Male
Asian	1.4078 ± 0.10769	1.5753 ± 0.12189
Black	1.3641 ± 0.10857	1.5296 ± 0.12255
White	1.4319 ± 0.11411	1.6144 ± 0.1277

**Figure 1:**
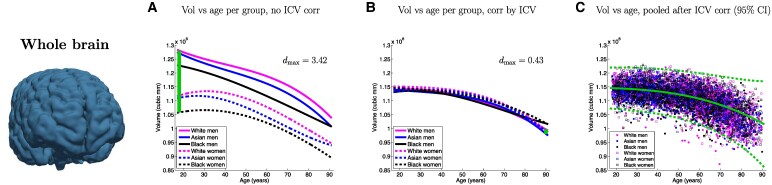
**Aging curves for the whole brain**. Aging curves for the whole brain and individual ROIs: comparison of sex- and race-specific trajectories before and after ICV correction, as well as trajectories from pooled, ICV-corrected data (95% confidence interval marked with dashed lines). The vertical lines represent the largest effect size (*d*_max_) across group pairs and ages (*N* = 5977).

**Figure 2:**
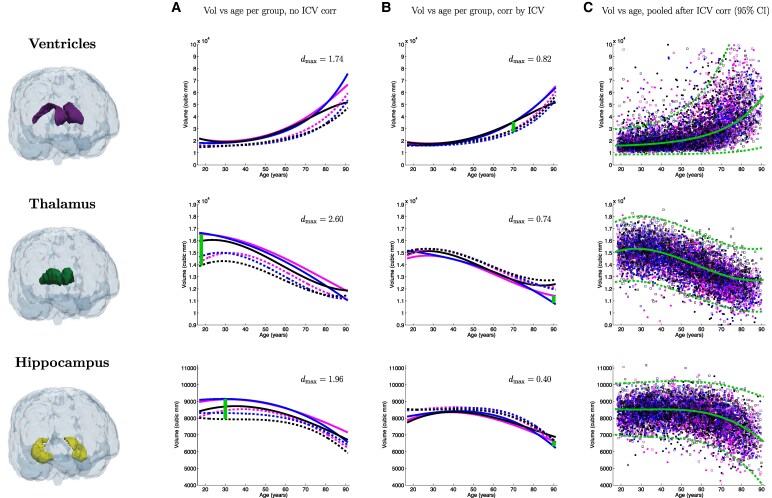
**Aging curves for the ventricles, thalamus and hippocampus.** Aging curves for the whole brain and individual ROIs: comparison of sex- and race-specific trajectories before and after ICV correction, as well as trajectories from pooled, ICV-corrected data (95% confidence interval marked with dashed lines). The vertical lines represent the largest effect size (*d*_max_) across group pairs and ages (*N* = 5977).

**Figure 3:**
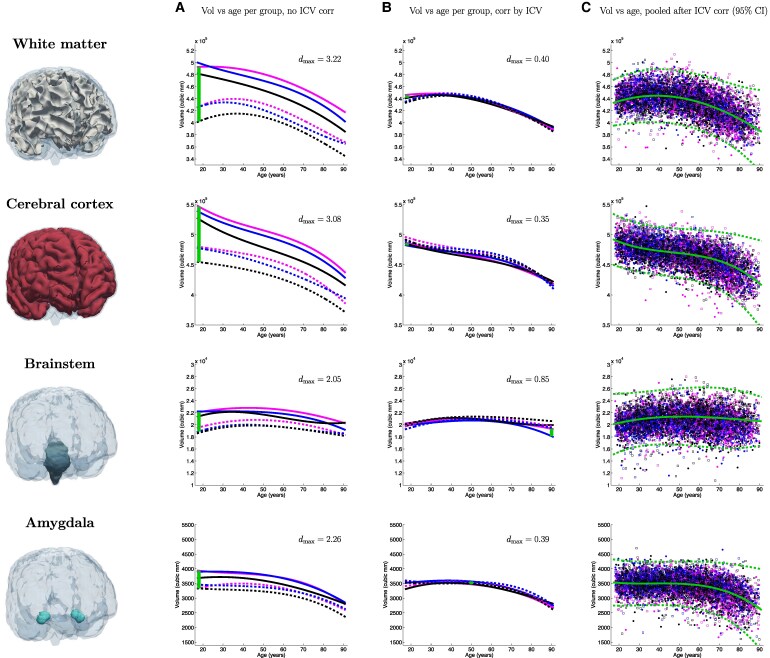
**Aging curves for the white matter, cerebral cortex, brainstem and amygdala**. Aging curves for the whole brain and individual ROIs: comparison of sex- and race-specific trajectories before and after ICV correction, as well as trajectories from pooled, ICV-corrected data (95% confidence interval marked with dashed lines). The vertical lines represent the largest effect size (*d*_max_) across group pairs and ages (*N* = 5977).

Figures 1–3 present a detailed analysis of sex- and race-specific aging trajectories before and after ICV correction. These figures also include effect sizes between groups, as well as aging trajectories using pooled, ICV-corrected data. We show trajectories for the volume of the whole brain, as well as for seven ROIs of interest: hippocampus, amygdala, ventricles, brainstem, thalamus, white matter and cortex. The curves before (Column A) and after (Column B) ICV normalization illustrate the powerful normalization properties of this correction, particularly for some ROIs such as the cortex, white matter, thalamus, or whole brain, which shift from strong differences to almost perfect overlaps. This is also reflected quantitatively in the effect sizes across race and sex, which are reduced by factors between 2 and 8. The pooled figures (Column C), which include the individual data points (with colour-coded race and sex), yield aging trajectories that are generally consistent with prior studies^14^—albeit with a much wider distribution of races. In the supplementary materials, we provide experimental results on additional ICV normalization methods, including division, residual, covariate and matching—following.^16-18^

## Discussion and conclusion

While most volumetric studies of brain ROIs may not thoroughly model ethnoracial factors, our results indicate that this omission may not undermine their results. The implementation of ICV correction—which is widespread in volumetric studies—seems sufficient to address most of the variations due to sex and race. By accounting for intracranial volume differences, ICV normalization effectively mitigates the confounding effects of head size, thereby enhancing the accuracy and comparability of neuroimaging studies across diverse populations. This adjustment not only improves the validity of comparative analyses but also enriches our understanding of how brain structures evolve with age across various demographic groups. It provides a more nuanced perspective on brain morphology by eliminating size-related biases, allowing researchers to focus on the actual structural differences and changes.

Nevertheless, our study has limitations, and further research is required to fully understand the broader implications of demographic factors on brain structure and function. One limitation is that our ‘ground truth’ racial labels are imperfect, due to incomplete data in the medical records, limitations in race self-reporting system (e.g. lack or subgroups or multi-racial options leading subjects of certain groups to more frequently select ‘other’), or failure of staff to appropriately inquire about race.^19^ To mitigate this noise in the racial labels, we used robust statistics based on the Laplace distribution, i.e. using medians instead of means (as Gaussians do). However, future work should use cleaner labels, if possible.

Additionally, we did not consider other potentially relevant confounders, such as years of education,^20^ socioeconomic status or the reason for MRI prescription; these were not consistently or reliably available for our retrospective cohort. Furthermore, the reason for MRI prescription is also highly impractical to model, due to its discrete, heterogeneous nature. Future work will assess whether ICV correction also handles these and other confounders. Further, it will be crucial to study a more comprehensive range of finer ROIs (e.g. cortical areas) and populations (e.g. multi-racial), affected by various diseases. Such analyses are needed to ensure that the findings in this study are applicable across even more diverse demographic groups—eventually leading to a more inclusive and equitable understanding of the human brain in health and in disease.

## Supplementary Material

fcaf271_Supplementary_Data

## Data Availability

Data from the MGB (Mass General Brigham) healthcare system cannot be shared due to privacy concerns.
