# Supplementary material for: The normalizing properties of intracranial volume across race and sex
Source: Brain Commun. 2025 Jul 16;7(4):fcaf271. doi: 10.1093/braincomms/fcaf271 (PMC12315546; doi:10.1093/braincomms/fcaf271)
Supplement: fcaf271_Supplementary_Data [file fcaf271_supplementary_data.pdf]

The normalizing properties of intracranial volume across race and sex (Additional Experiments)

This supplementary document includes additional experiments on various ICV normalization methods. In particular, we followed Nordenskjöld et al. [1] and Ardekani et al. [2], and considered additional ICV correction methods (Secs. 2-6) with a non-parametric spline model for age and a robust L1 / Laplacian model.

1 Naming convention

- a_n = age of subject n
- v_n = ROI volume of subject = n
- ICV_n = ICV of subject = n
- $\overline{ICV}$ = average ICV of the cohort.
- $\mu(a_n; \theta_\mu)$: location at age a_n, parameterized by θ_μ.
- $b(a_n; \theta_b)$: scale at age a_n, parameterized by θ_b.
- ϵ_n: residual / error of subject n after modeling. This is a random variable that follows a Laplacian distribution centered at zero and scale $b(a_n; \theta_b)$, i.e., it follows: $\epsilon_n \sim \text{Laplace}[0, b(a_n; \theta_b)]$.

2 Method 1: bspline alone without ICV correction (included in original submission)

$$v_n = \mu(a_n; \theta_\mu) + \epsilon_n,$$

or, equivalently,

$$v_n \sim \text{Laplace}[\mu(a_n; \theta_\mu), b(a_n; \theta_b)].$$

3 Method 2: division (included in original submission)

$$v_n \frac{\overline{ICV}}{ICV_n} = \mu(a_n; \theta_\mu) + \epsilon_n,$$

or, equivalently,

$$v_n \sim \text{Laplace}\left[\frac{ICV_n}{\overline{ICV}} \mu(a_n; \theta_\mu), \frac{ICV_n}{\overline{ICV}} b(a_n; \theta_b)\right].$$

New results are shown in Fig. 1.

4 Method 3: residual

$$v_n = \beta_{ICV}(ICV_n - \overline{ICV}) + \mu(a_n; \theta_\mu) + \epsilon_n,$$

or, equivalently,

$$v_n \sim \text{Laplace}[\mu(a_n; \theta_\mu) - \beta_{ICV}(ICV_n - \overline{ICV}), b(a_n; \theta_b)].$$

New results are shown in Fig. 2.

5 Method 4: covariate

$$\begin{aligned} v_n = &\beta_{sex}[\delta(sex_i = male) - (1/2)))] \\ &+\beta_{asian}[\delta(race_i = asian) - (1/3)] \\ &+\beta_{white}[\delta(race_i = white) - (1/3))] \\ &+\beta_{ICV}(ICV_n - \overline{ICV}) \\ &+\mu(a_n; \theta_\mu) + \epsilon_n, \end{aligned}$$

or, equivalently,

$$\begin{aligned} v_n \sim \text{Laplace}[&\mu(a_n; \theta_\mu) - \beta_{ICV}(ICV_n - \overline{ICV}) \\ &- \beta_{sex}[\delta(sex_i = male) - (1/2)))] \\ &- \beta_{asian}[\delta(race_i = asian) - (1/3)] \\ &- \beta_{white}[\delta(race_i = white) - (1/3))], \\ &b(a_n; \theta_b)]. \end{aligned}$$

Here, we assume that the "default" sex is female and the "default" race is black; their intercepts are absorbed by μ. Note that we demean the indicator variables by their expectations, too: (1/2) for sex, (1/3) for race. Note that, for this method, after fitting the model, we *do not* correct for race or sex, to keep visualizing potential ICV differences due to these covariates – i.e., we plot $\mu(a_n; \theta_\mu) - \beta_{ICV}(ICV_n - \overline{ICV})$.

New results are shown in Fig. 3.

6 Method 5: matching

This method follows Ardekani et al. [2], which presents a greedy approach that matches subjects with very similar ICV, until we cannot find any more pairs with ICV differences lower than 1,000 mm^3.

Note that since we have 6 groups (3 races times 2 sexes), we use an average distance as a metric to threshold "acceptable" sextuples, which yields 1,644 subjects, i.e., losing 72.5% of the total subjects. This large data loss due to the relatively high number of groups leads to noisy results, which we are including in this letter of response (for completeness), but not in the manuscript.

New results are shown in Fig. 4.

7 Summary of results

The choice of ICV normalization methods has very little influence on the correction, and no impact on the conclusion of our study. Correctiong by division and regression yield maximum effect sizes ($d_{\max}$) that are within 0.2 of each other for all ROIs (and below 0.1 for all but one ROI). When sex and race are included covariates, the results are almost exactly the same as when correcting by regression. As mentioned above, the matching algorithm leads to a strong loss of data which in turns yields noisier results – but even those produce substantial reductions in $d_{\max}$.

References

[1] Nordenskjöld, R., Malmberg, F., Larsson, E.-M., Simmons, A., Ahlström, H., Johansson, L., Kullberg, J.: Intracranial volume normalization methods: considerations when investigating gender differences in regional brain volume. Psychiatry Research: Neuroimaging **231**(3), 227–235 (2015)

[2] Ardekani, B.A., Figarsky, K., Sidtis, J.J.: Sexual dimorphism in the human corpus callosum: an mri study using the oasis brain database. Cerebral cortex **23**(10), 2514–2520 (2013)

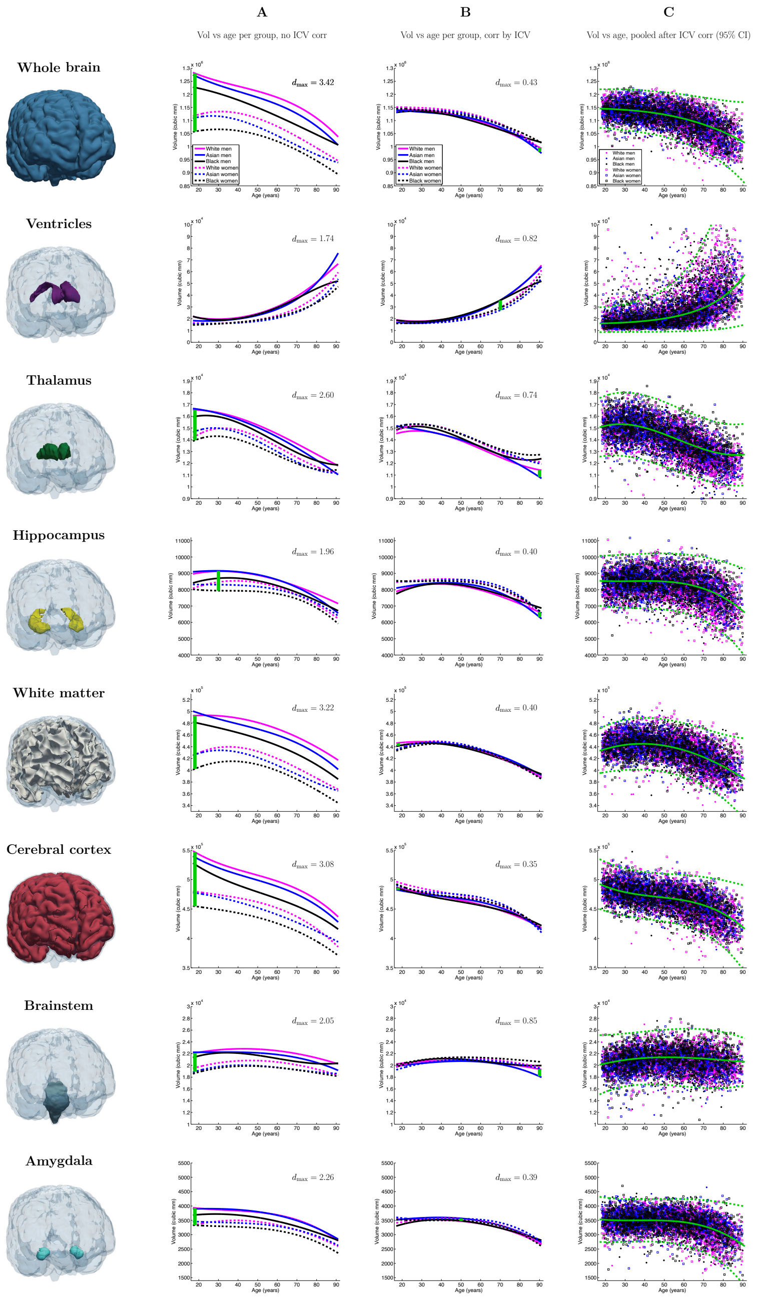

Supplementary Fig. 1 Aging curves for the whole brain and individual ROIs: comparison of sex- and race-specific trajectories before and after ICV correction (Sec. 3: Method 2 - division), as well as trajectories from pooled, ICV-corrected data (95% confidence interval marked with dashed lines). The vertical green lines represent the largest effect size ($d_{\max}$) across group pairs and ages. (N=5,977)

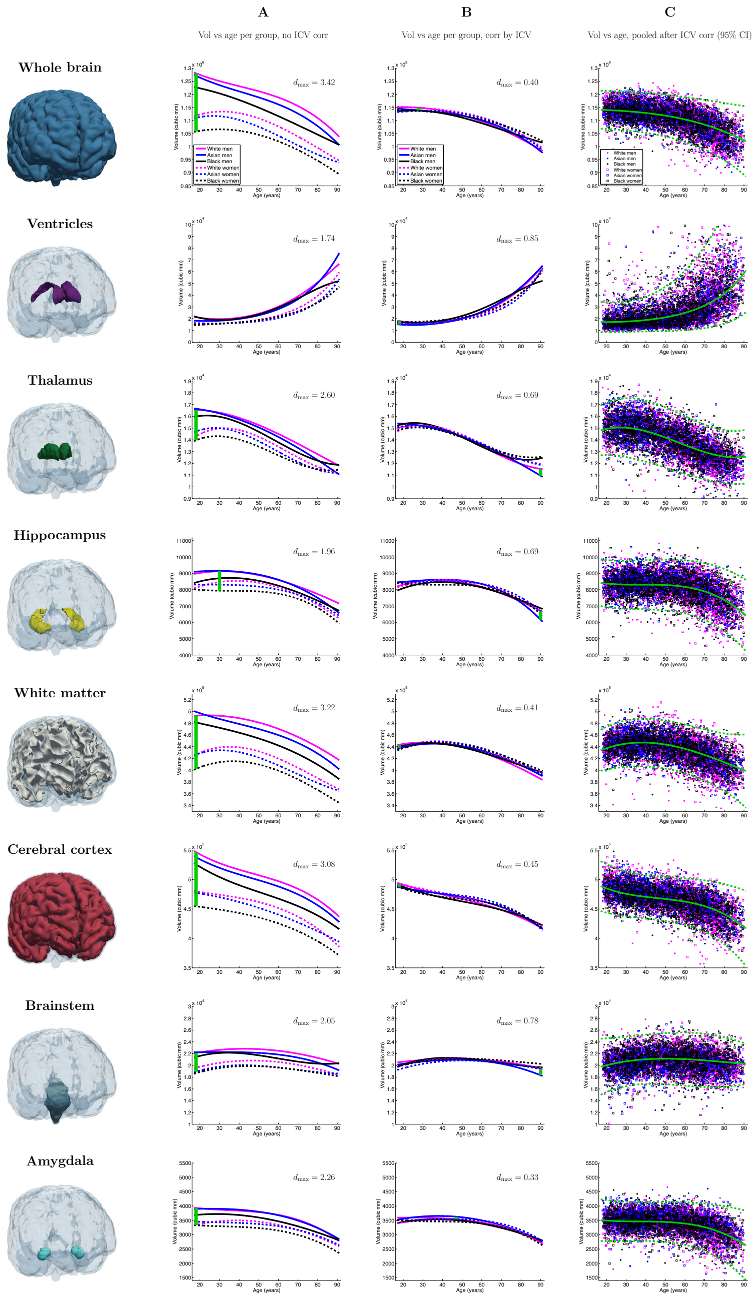

Supplementary Fig. 2 Aging curves for the whole brain and individual ROIs: comparison of sex- and race-specific trajectories before and after ICV correction (Sec. 4: Method 3 - residual), as well as trajectories from pooled, ICV-corrected data (95% confidence interval marked with dashed lines). The vertical green lines represent the largest effect size ($d_{\max}$) across group pairs and ages. (N=5,977)

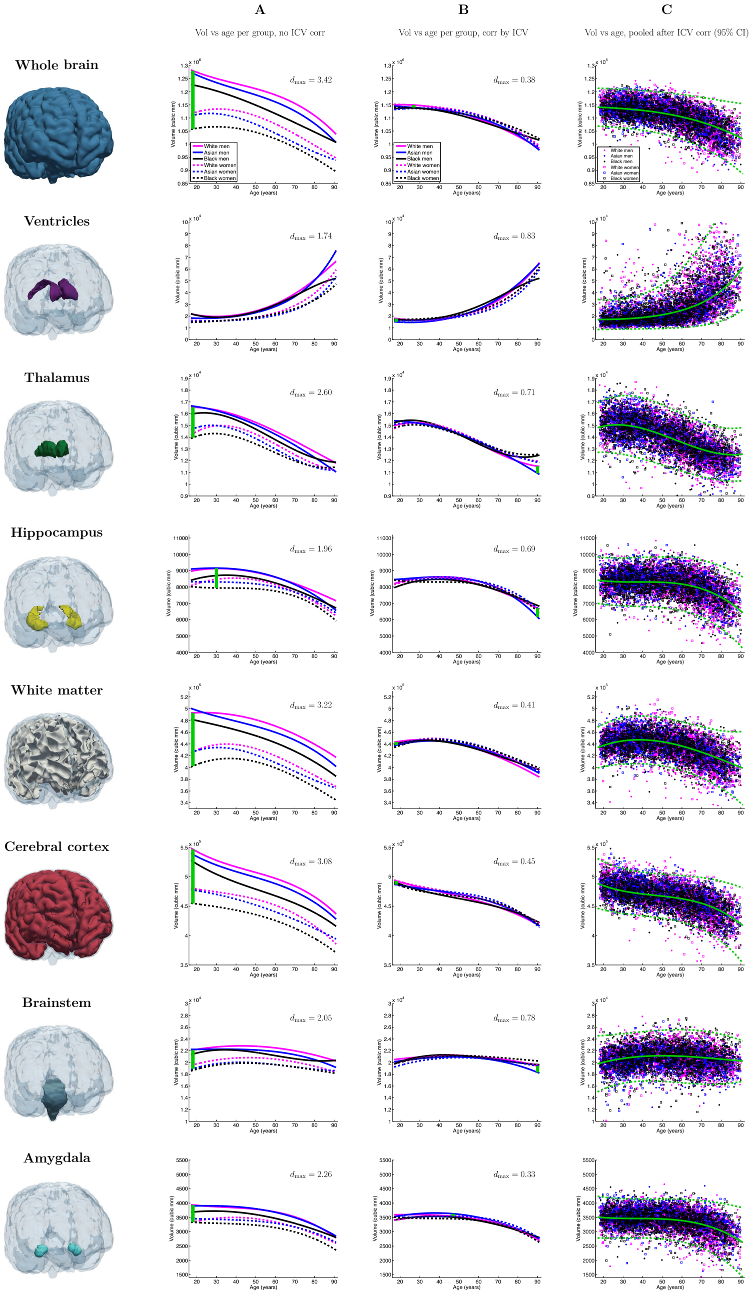

Supplementary Fig. 3 Aging curves for the whole brain and individual ROIs: comparison of sex- and race-specific trajectories before and after ICV correction (Sec. 5: Method 4 - covariate), as well as trajectories from pooled, ICV-corrected data (95% confidence interval marked with dashed lines). The vertical green lines represent the largest effect size ($d_{\max}$) across group pairs and ages. (N=5,977)

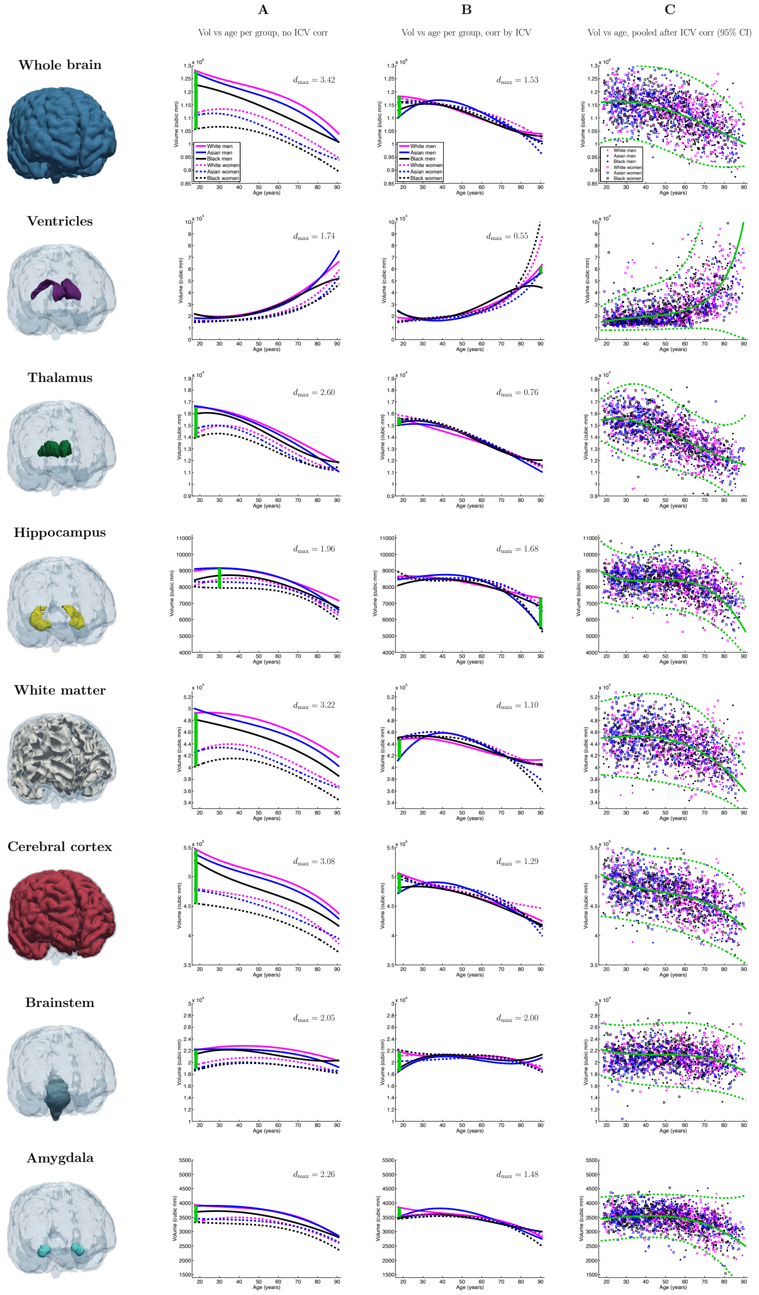

Supplementary Fig. 4 Aging curves for the whole brain and individual ROIs: comparison of sex- and race-specific trajectories before and after ICV correction (Sec. 6: Method 5 - matching), as well as trajectories from pooled, ICV-corrected data (95% confidence interval marked with dashed lines). The vertical green lines represent the largest effect size ($d_{\max}$) across group pairs and ages. (N=5,977)
